# Iterleukin 1 alpha is a marker of endothelial cellular senescent

**DOI:** 10.1186/1742-4933-3-4

**Published:** 2006-04-06

**Authors:** Massimo Mariotti, Sara Castiglioni, Daniela Bernardini, Jeanette AM Maier

**Affiliations:** 1 Department of Preclinical Sciences, University of Milan Medical School, Via GB Grassi, 74 Milan, Italy

## Abstract

**Background:**

The functional changes associated with endothelial senescence may be involved in human aging and age-related vascular disorders. Since the inflammatory cytokine interleukin (IL-)1 inhibits endothelial growth, we evaluated the expression of IL-1α, IL-1β and their antagonist, the IL-1 receptor antagonist (IL-1ra), in endothelial in vitro senescence and quiescence. We also examined the expression of IL-1α in human senescent and progeric fibroblasts.

**Results:**

We found that the overexpression of IL-1α specifically characterizes endothelial senescence. No modulation of this cytokine was observed in endothelial quiescence and in senescent or progeric human fibroblasts. The expression of IL-1β and IL-1ra was also assessed and found not to be affected by senescence.

**Conclusion:**

Our results indicate that a dysfunction of the cytokine network associates with aging and point to a specific role of IL-1α in endothelial senescence.

## Introduction

The endothelium is a dynamic, heterogeneous, disseminated organ that possesses vital secretory, synthetic, metabolic and immunologic functions. *In vivo *endothelial cells (EC) represent a large population of quiescent cells lining the vessels. Macrovascular EC rarely divide with a turnover rate of approximately once every three years [1], although replication is increased under conditions that favor atherogenesis, such as hypertension, high cholesterol levels and anatomical branch points. In vitro, EC have a finite number of cell replication reaching replicative senescence [2]. This cessation of cell division is accompanied by a specific set of changes in cell function, morphology and gene expression [3,1] that may contribute to age-associated diseases, including atherosclerosis. Interestingly, vascular endothelial cells with senescence-associated phenotypes have been detected in the atherosclerotic regions of human aorta [4] and coronary arteries [5]. Accordingly, multiple baloon endothelial denudation in non-atheromatous rabbit carotid arteries promoted the accumulation of senescent cells in the arterial wall [6].

Endothelial senescence is modulated in part by the inflammatory cytokine interleukin (IL-)1α [2,7]. IL-1 and its family members are expressed in human atherosclerotic vessels, mainly in the endothelium [8]. It is noteworthy that EC replicative senescence and IL-1 have been associated with atherosclerosis.

Although senescence and quiescence have a common denominator represented by the inhibition of cell growth, the two processes are different, because senescence is characterized by an irreversible growth arrest as well as by a specific gene expression profile [3].

This paper addresses the relation between IL-1α, IL-1β and IL-1ra expression and macrovascular endothelial cell quiescence and senescence. We also examined the expression of IL-1α in human senescent and progeric fibroblasts. We conclude that the overexpression of IL-1α is a specific marker of in vitro endothelial senescence.

## Methods

### Cell culture

Human umbilical vein EC (HUVEC) were cultured in M199 containing 10% fetal calf serum (FCS), Endothelial Cell Growth Supplement (150 μg/ml) and heparin (5 U/ml) on 2% gelatin coated dishes. All culture reagents were from Gibco. Human fibroblasts from progeric individuals (GM0498, 3 year-old male; GM2037, 13 year old male) and age-matched controls (AG6917, 3 year-old male; AG3513, 13 year old male) were from ATCC and cultured in D-MEM containing 20% FCS. Human dermal fibroblasts were isolated and propagated in D-MEM with 10% FCS until they reached cellular senescence [9].

The population doublings (PD) were calculated as log_2 _(number of cells at time of subculture/number of cells plated). The senescent phenotype was assessed by evaluating the senescence-associated (SA)-beta galactosidase activity as described [10].

### Northern blot and RT-PCR analysis

HUVEC were rinsed with phosphate buffered saline and lyzed in RNAzol (Gibco). PolyA^+ ^RNA was purified on oligodT columns, electrophoresed on a 1% agarose gel containing 2.2 M formaldehyde, capillary blotted onto nylon membranes and UV crosslinked. IL-1α and GAPDH cDNAs were labelled with a random primer labeling kit (Ambion). Filters were hybridized in 0.5 M sodium phosphate (pH 7.2) containing 7% SDS, 1 mM EDTA and 20% formamide at 65°C for 20 h and extensively washed at high stringency before autoradiography. The results were quantitated by densitometry. To establish whether comparable amounts of RNA had been loaded, the ratio GAPDH/IL-1α was evaluated. For RT-PCR, 1 μg of total RNA was reverse transcribed and PCR amplification was carried out using 1/50 of the final RT reaction. Each amplification cycle consisted of 30 sec at 95°C, 30 sec at 52°C and 1 min at 72°C using 30 pmol of each primer. The reaction was stopped after 15 or 30 cycles. One fifth of the reaction mix was separated on a 1% agarose gel. The primers used to amplify IL-1β are the following: 5'-GACTTGTTCTTTGAAGTCGAT-3' (sense) and 5'-TAGAGTGGGCTTATCATCTTT-3' (reverse). The primers for IL-1ra are: 5'-ATGGAAATCTGCAGAGGCCTCCGCAGT-3' (sense) and 5'-CTGGTCAGCTTCCATCGCTGTGCAGAGGAA-3' (antisense). The sequence of the GAPDH primers has been published [2].

### Western blot

HUVEC were lysed in 10 mM Tris-HCl (pH 7.4) containing 3 mM MgCl_2_, 10 mM NaCl, 0,1% SDS, 0,1% Triton X-100, 0,5 mM EDTA and protein inhibitors, separated on 15% SDS-PAGE and transferred to nitrocellulose sheets. Western blot analysis was performed using polyclonal goat antibodies against IL-1α (Santa Cruz-TebuBio). Secondary antibodies were labeled with horseradish peroxidase (Amersham Pharmacia Biotech). The SuperSignal chemiluminescence kit (Pierce) was used to detect immunoreactive proteins following the manufacturer's instructions. The blots were stripped and incubated with an anti-actin antibody (Santa Cruz – Tebu-bio) to show that comparable amounts of protein were loaded per lane. Densitometric analysis was performed to better quantitate the results. All the western blots have been repeated at least three times on cell extracts from different experiments.

## Results

### IL-1α levels in proliferating vs quiescent HUVEC

**Figure 1:**
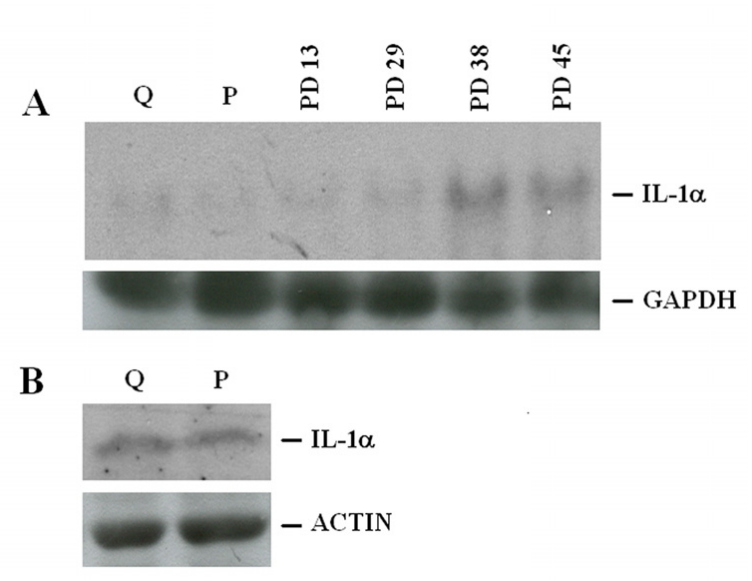
**IL-1α expression in HUVEC**. (A) 3 μg of polyA^+ ^RNA were utilized for Northern analysis. GAPDH hybridization shows that comparable amounts of RNA were loaded per lane. P: proliferating cells (PD 20); Q: quiescent cells (PD 20). PD indicates the number of population doublings undergone by HUVEC (from young -PD 13- to senescent – PD 45-). (B) Cell lysates (450 μg) from proliferating vs quiescent HUVEC were separated by a 15% SDS-PAGE and western blot was performed as described in the methods. After stripping, the blot was incubated with an anti-actin antibody to show that comparable amounts of protein were loaded per lane. P: proliferating cells; Q: quiescent cells, both at PD 20.

On culture plates, HUVEC grow until they form a perfect monolayer. At this stage, cells stop growing and become quiescent. This pattern correlates with the arrest of thymidine incorporation (not shown). Since IL-1α inhibits endothelial proliferation, we evaluated whether IL-1α was modulated in actively proliferating versus quiescent HUVEC, both at PD 20. PolyA^+ ^RNA was separated by electrophoresis and Northern blot was performed. We detected no modulation of IL-1α RNA in quiescent vs proliferating cells (Fig. 1A). Accordingly, the protein levels of IL-1 were comparable in quiescent and proliferating cells (Fig. 1B). Similar results were obtained in HUVEC cells when quiescence was reached by growth factor withdrawal (not shown).

### IL-1s expression in HUVEC cells at various PD

**Figure 2:**
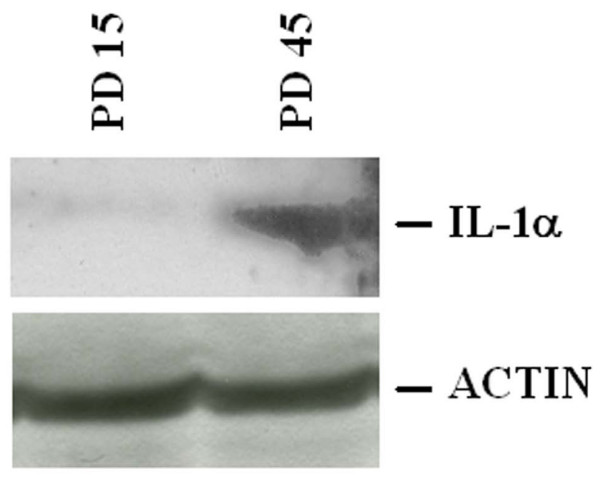
**IL-1α total amounts in young and senescent HUVEC**. Cell lysates (350 μg) from young and senescent HUVEC were separated by a 15% SDS-PAGE and western blot was performed as described. After stripping, the blot was incubated with an anti-actin antibody to show that comparable amounts of protein were loaded per lane. PD 15: young HUVEC; PD 45: senescent HUVEC.

Since cellular senescence correlates with the decline of the proliferating rate and an antisense against IL-1α extends endothelial lifespan [2], we evaluated IL-1α levels in HUVEC at different PD. As shown in figure 1A, a gradual increase of IL-1α mRNA paralleled the increase of PD, as detected by Northern blot performed on polyA^+ ^RNA. Accordingly, we detected higher amounts of IL-1α in senescent (PD 45) than in young (PD 15) cells by western analysis (Fig. 2).

**Figure 3:**
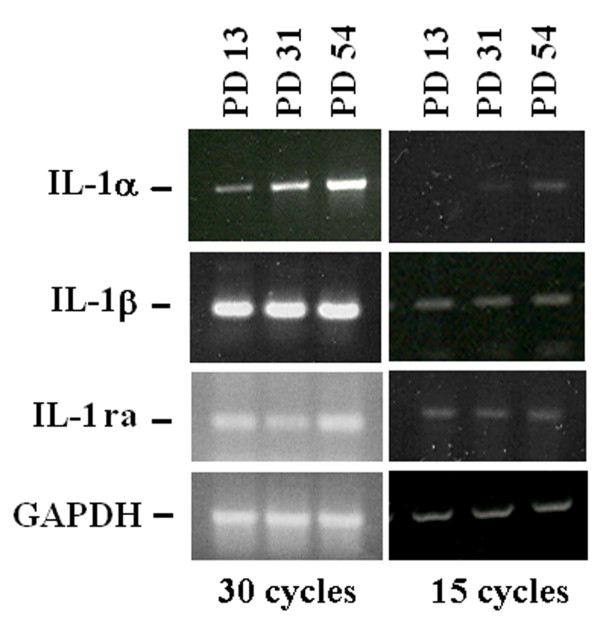
**IL-1α, IL-1β and IL-1ra expression in young and senescentHUVEC**. RNA was extracted from HUVEC at different PD (13, 31,54). RT-PCR was performed using specific primers for IL-1α, IL-1β and IL-1ra. PCR amplification was stopped after 15 and 30 cycles to provide a better quantification of the results. GAPDH was utilized to show that similar amounts of RNA had been reverse-transcribed.

Because i) IL-1α shares many activities with IL-1β and ii) their activities are antagonized by the IL-1 receptor antagonist (IL-1ra), we determined the expression of the members of the IL-1 family, i.e. IL-1α, IL-1β and IL-1ra by RT-PCR in HUVEC at different PDs. To provide a better quantification, the PCR reaction was stopped after 15 or 30 cycles. In both conditions, we found no significant modulation of the total amounts of IL-1β and IL-1ra in young and senescent endothelial cells, while we confirmed the induction of IL-1α expression (Fig. 3).

### IL-1s in young, senescent and progeric human fibroblasts

**Figure 4:**
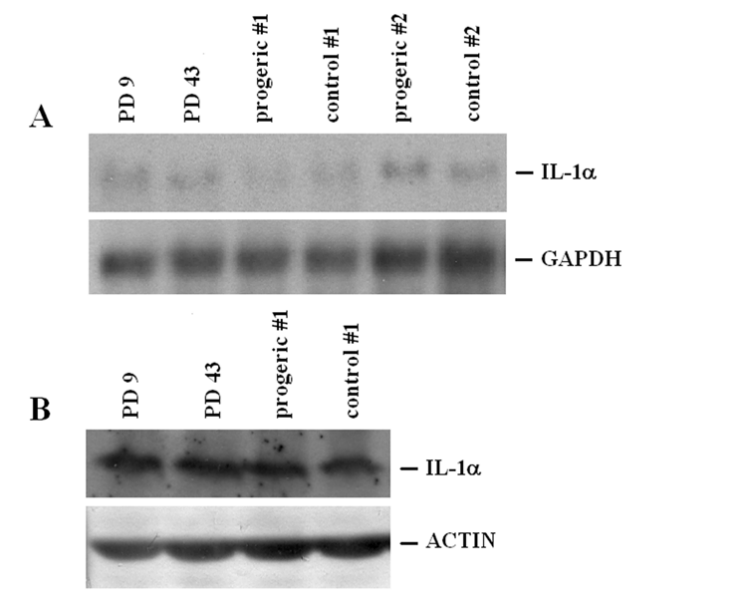
**IL-1α expression in young, senescent and progeric human dermal fibroblasts**. (A) 3 μg of polyA^+ ^RNA were utilized for Northern analysis. GAPDH hybridization shows that comparable amounts of RNA were loaded. PD 9: young fibroblasts; PD 43: senescent fibroblasts. Two different progeric cell types and age-matched controls have been utilized as described. Progeric and control #1: GM0498 and AG6917, respectively; progeric and control #2: GM2037 and AG3513, respectively. (B) Cell lysates (250 μg) from young, senescent and progeric (#1: GM0498 and AG6917) dermal fibroblasts were separated by a 15% SDS-PAGE and western blot was performed. After stripping, the blot was incubated with an anti-actin antibody to show that comparable amounts of protein were loaded per lane. PD 9: young fibroblasts; PD 43: senescent fibroblasts; progeric and control #1: GM0498 and AG6917.

To determine whether the relationship between IL-1s and senescence occurred also in fibroblasts, we examined young and senescent human dermal fibroblasts for their expression of IL-1α by Northern on polyA^+ ^RNA and western blot and found no differences (Fig. 4A and 4B). Progeria is a rare premature aging syndrome which is characterized by external stigmata of aging and death within the fierst two decades of life [11]. We therefore evaluated IL-1α expression in dermal fibroblasts derived from progeric individuals. Figure 4A and 4B show that IL-1α mRNA and protein levels are not significantly modulated in progeric vs age-matched dermal fibroblasts.

**Figure 5:**
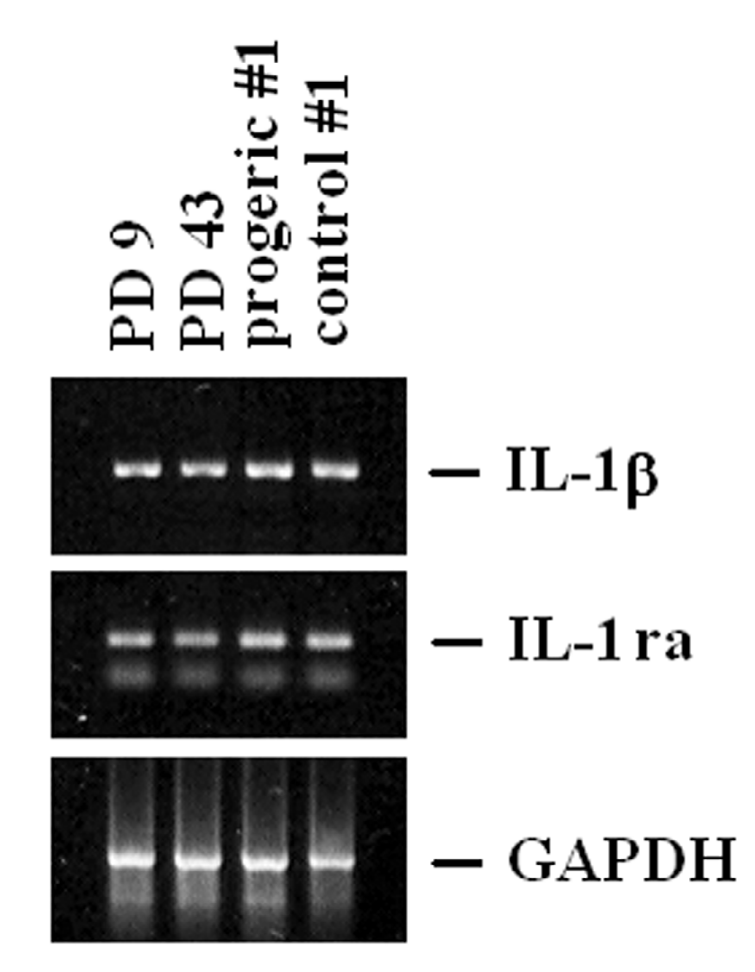
**IL-1β and IL-1ra expression in young, senescent and progeric human dermal fibroblasts**. RNA was extracted to perform an RT-PCR using specific primers for IL-1β and IL-1ra. GAPDH was utilized to show that comparable amounts of RNA had been reverse-transcribed. 30 cycles of PCR were performed. PD 9: young fibroblasts; PD 43: senescent fibroblasts; progeric and control #1: GM0498 and AG6917.

By RT-PCR, we show that no modulation occurs in the steady state levels of IL-1β and IL-1ra (Fig. 5) in senescent and progeric fibroblasts.

## Discussion

The results of the present study indicate that the overexpression of IL-1α specifically characterizes endothelial senescence. No modulation of this cytokine was observed in endothelial quiescence and in senescent or progeric human fibroblasts.

IL-1α shares many activity with IL-1β, since they act by binding to a common receptor, the type I IL-1 receptor [12]. A third member of the family, the IL-1 receptor antagonist (IL-1ra), also binds to the type I IL-1 receptor and blocks the receptor, preventing the action of the agonist IL-1s [12]. In senescent endothelial cells we did not detect any modulation of the mRNA levels for IL-1β and IL-1ra. We therefore propose that IL-1α could be used as a marker of endothelial senescence. IL-1α causes multiple responses in vascular endothelial cells including inhibition of cell proliferation [13], induction of adhesion molecules which bind leukocytes [14] and promotion of thrombus formation [15]. Indeed, *in vitro*, IL-1α overexpression has been linked to endothelial lifespan and to several dysfunctions [2]. It is noteworthy that senescent endothelial cells are found on the surface of atherosclerotic plaques [5] and that IL-1 is produced by endothelium on aged coronary arteries [8].

Increased vascular production of pro-inflammatory cytokines may contribute to the increased plasma levels of these mediators in aging [16,17]. We propose that the upregulation of IL-1 can be linked to the activation of various pathophysiological programs that underlie the complex biological phenomenon described as "vascular aging". Proinflammatory status of aged arteries may shift the intravascular environment from a hemodynamically stable state to a pro-coagulant, pro-oxidant state which may favour an exaggerated response to vessel injury, promoting the development of ischemic heart disease in the elderly.

IL-1ra allelic polymorphisms affect replicative lifespan of human EC [10]. The polymorphism IL-1RN*2*2, which decreases the levels of IL-1ra, was associated with increased numbers of senescent endothelial cells and an inhibition of proliferation, while the addition of IL-1ra restored the proliferative potential of the cells and extended their lifespan [10]. Because endothelial turnover is enhanced under conditions that favour atherogenesis thus leading to a senescent phenotype, it is noteworthy that the IL-1RN*2 allele is associated with atherosclerotic coronary disease [18]. All together, these data suggest that IL-1ra may prevent the senescent-promoting effects of IL-1 in the endothelium.

We did not detect any modulation of the steady state levels of IL-1s in *in vitro *aged human dermal and in progeric fibroblasts. This is in agreement with a previous study performed on IMR-90 cells, demonstrating that the human diploid fibroblast senescence pathway is independent of IL-1α mRNA levels [19]. On the contrary, expression of IL-1β and IL-1ra was induced in senescent mouse embryonic fibroblasts [20]. Interestingly, the IL-1ra^-/- ^mice presented early mortality compared to wild-type mice and accelerated senescence was observed in IL-1ra deficient fibroblasts [21]. All together these data indicate that a dysfunction of the cytokine network associates with aging and point to a specific role of IL-1α in endothelial senescence.

## Abbreviations

interleukin-1 IL-1

IL-1 receptor antagonist IL-1ra

endothelial cells EC

human umbilical vein EC HUVEC

reverse transcription- polymerase chain reaction RT-PCR
